# The Investigation of Rotary Bending Fatigue Properties of 4Cr14Ni14W2Mo Engine Valve Steel Processed by Surface Mechanical Rolling Treatment

**DOI:** 10.3390/ma19010078

**Published:** 2025-12-25

**Authors:** Ge Sun, Zhifeng Liu, Zengrui Yuan, Rong Qu, Fuqiang Lai

**Affiliations:** 1 Huaiji Dengyue Valve Co., Ltd., Zhaoqing 526400, China; sunge@huaijivalve.com (G.S.); rongqu@huaijienginevalve.com (R.Q.); 2 School of Mechanical Engineering and Automation, Fuzhou University, Fuzhou 350116, China; zhifeng_liu666@163.com; 3 Weichai Power Co., Ltd., Weifang 261061, China; yuanzr@weichai.com

**Keywords:** 4Cr14Ni14W2Mo engine valve steel, surface mechanical rolling treatment, surface modified layer, rotary bending fatigue, failure mechanism

## Abstract

In order to address potential fatigue fractures at the valve stem-neck junction during engine operations, surface mechanical rolling treatment (SMRT) was introduced to enhance the rotary bending fatigue (RBF) performance of 4Cr14Ni14W2Mo engine valve steel in this study. The results indicate that the increasing number of rolling passes induces a modified surface layer characterized by refined grains and dislocations, increased hardness, and compressive residual stress (RS). SMRT specimens exhibited improved tensile strength but plasticity performance was decreased. At room temperature (RT) about 25 °C, the fatigue limit at 1 × 10 _7_ cycles of specimens treated with 10 rolling pass was increased from 437 MPa to 613 MPa (40.3%). At 400 °C, the fatigue limit of specimens treated with 10 passes was increased from 376 MPa to 425 MPa (13.0%) at 400 °C, but decreased at 650 °C. The enhanced fatigue performance is attributed to a modified surface layer, leading to the shift of the crack initiation to the subsurface. However, excessive rolling passes and high temperature (650 °C) significantly reduce the material plasticity, accelerating crack initiation and propagation, thus compromising performance.

## 1. Introduction

The internal combustion engine serves as the vital core of heavy industrial products such as automobiles and steamship. Among its critical components, the valves have significant influence on the reliability and efficiency of the engine. Engines operate within high-temperature and high-pressure environments, particularly exhaust valves would endure temperatures ranging from 600 °C to 800 °C [1]. Additionally, further increases in engine emission standards and the need for lightweight, high-power engines mean that valves need to perform a better performance and obtain a longer service life [2,3]. There are three main forms of valve failure: (1) fatigue fracture failure of valve neck or stem, (2) wear failure of valve sealing interface or stem, (3) burn failure of valve disc.

**Figure 1:**
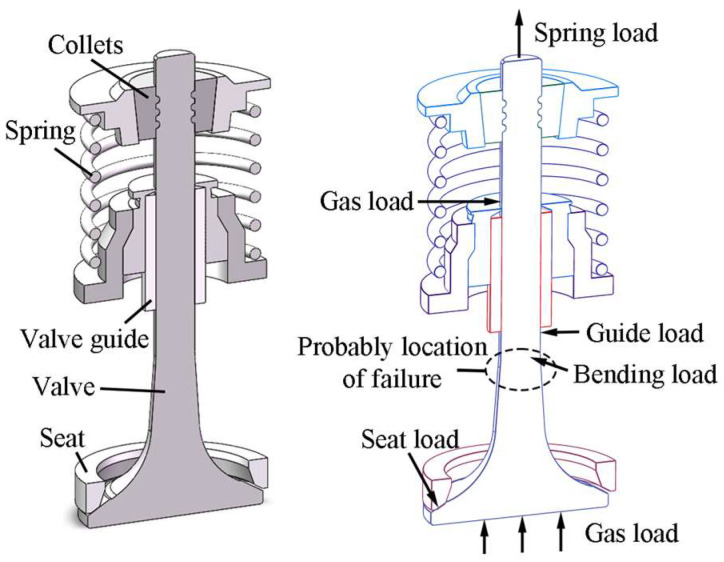
Unilateral valve seating in an internal combustion engine.

Ideally, the valve and valve seat insert would mate perfectly, with the valve only experiencing tension and compression forces. However, due to friction loss, thermal distortion, assembly discrepancies, part inaccuracies, and other factors, the axis of the valve and valve seat insert may become misaligned. This results in imperfect fit between the valve sealing interface and valve seat insert, leading to unilateral valve seating as shown in Figure 1. Unilateral valve seating results in an asymmetrical impact force on the valve sealing surface, inducing an extra bending moment on the valve stem [4]. The transition position between the valve stem and neck becomes a vulnerable zone susceptible to bending fatigue fractures [5]. Fatigue fracture failures occurrences within the valve neck-stem junction can be attributed to the cumulative effects of long-term cyclic bending stresses. If the valve breaks during engine operating conditions, the valve head would fall into the engine cylinder, colliding with the piston and causing the engine to stop running, resulting in severe consequences. Therefore, the durability research of the valve material is crucial for ensuring the reliability of engine valve operation.

Surface modification techniques are commonly used to improve the material fatigue resistance, including ultrasonic surface rolling [6], nitriding [7], shot peening [8], PVD coatings [9] and so on. One of such methods is surface mechanical rolling treatment (SMRT), which effectively processes axial workpieces, such as the potential failure location of engine valves above mentioned. SMRT applies external forces to introduce severe elastic-plastic deformation on the surface of workpiece, generating a gradient fine structure (GFS) layer on the surface, where the average grain size gradually increases from the nanoscale to sub-micron or micron scale from surface to subsurface. The GFS layer can significantly improve the metallic fatigue performance [10,11,12,13,14]. For example, Dong et al. [10] achieved a 700 μm thick GFS layer on rare earth addition bearing steel Re-GCr15 by using SMRT, increasing fatigue limit by 14.3%. Carneiro et al. [11] produced a 300 μm thick GFS layer on 316L stainless steel through SMRT, significantly improving its fatigue performance. Zhang et al. [12] prepared GFS on the 17Cr2Ni2MoVNb alloy steel surface through ultrasonic surface rolling, and the rolling contact fatigue resistance was experimentally proven to have been improved. According to the reports from Martins et al. [13], deep rolling promoted a reduction in surface roughness, promotes surface deformation, and caused an increase in both surface hardness and fatigue life of AISI 4140 steel. In addition, Zhang et al. [14] introduced gradient grain layer to the 7075-T651 aluminum alloy surface by means of SMRT. It is found that fatigue crack initiation mode exhibited the transition from surface to interior as the stress decreases after SMRT. They also discussed the fatigue strengthening mechanism based on the GFS layer generated after SMRT. The fatigue strengthening mechanism could be attributed a combination of GFS layer and the transition of crack initiation mode. These findings could be owed to the fact that GFS layer can suppress the initiation and propagation of surface cracks.

Previous studies on the influence of SMRT parameters have observed that higher forces typically result in increased hardness and compressive residual stress (RS) in the surface layer of material [15,16]. However, most of the studies ignored the effect of the number of rolling passes.

**Figure 2:**
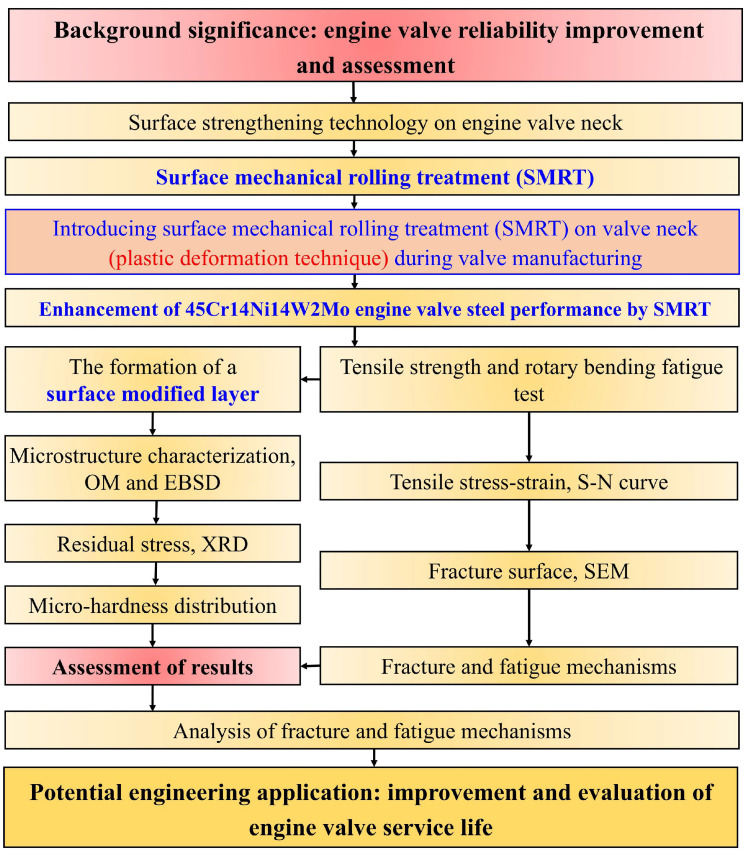
Methodology flowchart of the study.

In this study, a surface modified layer was generated on 4Cr14Ni14W2Mo steel by using SMRT. Subsequently, tensile tests and rotary bending fatigue (RBF) tests were conducted on both untreated and SMRT treated specimens, and the related fracture and fatigue mechanisms were analyzed. The methodology flowchart of this study is shown in Figure 2. The outcome of this study includes potential engineering applications: improvement and evaluation of engine valve service life.

## 2. Experimental Details

### 2.1. Specimen Preparation by Double-Sided Support Auxiliary Device

**Figure 3:**
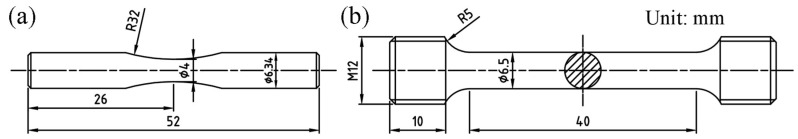
Schematic of the specimen geometry: (**a**) RBF, (**b**) tensile.

**Figure 4:**
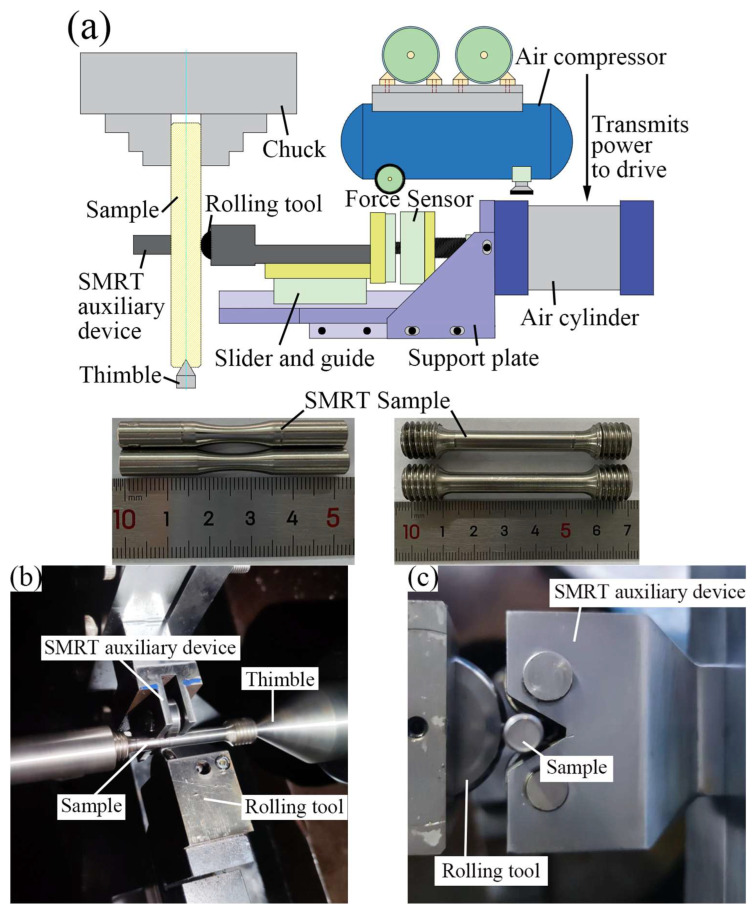
SMRT double-sided support device: (**a**) schematic diagram, (**b**,**c**) SMRT double-sided support auxiliary device.

The chemical compositions of the received 4Cr14Ni14W2Mo steel bars are presented in Table 1. Based on relevant Chinese national testing standard [17], the specimen geometry with hourglass shape for the RBF tests is determined, as shown in Figure 3a. Furthermore, according to the Chinese national testing standard [18], the specimen geometry with dog-bone shape for the tensile tests is determined, as shown in Figure 3b. Initially a Φ55 × 300 mm bar was wire-cut into bar blanks for RBF (Φ7.2 × 120 mm) and tensile (Φ11 × 80 mm) tests. After quenching at 820 °C for 30 min with water cooling, the bar blanks were machined by using computer numerical control lathe and electro-spark discharge machining to obtain the specimens used in the RBF tests and tensile tests. Subsequently, SMRT was conducted by using a CA6140 lathe equipped with a specially designed double-sided support auxiliary device which is shown in Figure 4. Static pressure was meticulously controlled by using an air compressor, air valves, force sensors, and pneumatic cylinder. An turn-assisted double-sided auxiliary device was specifically designed for SMRT to mitigate potential adverse effects of unilateral rolling, such as specimen bending [19,20]. Specially, a smooth and rotatable rolling tool was pressed at a specified force into the specimen surface and moved along the specimen at a speed of Vr, while the specimen rotated at a speed of Vs. Furthermore, lubricant of Mobil 1^TM^ 0W-40 Machine Oil (Exxon Mobil Corporation, Houston, TX, USA) was sprayed onto the rolling tool and specimen surface during SMRT to facilitate lubrication and prevent overheating. SMRT processing parameters are detailed in Table 2.

### 2.2. Rotary Bending Fatigue Test Details

**Figure 5:**
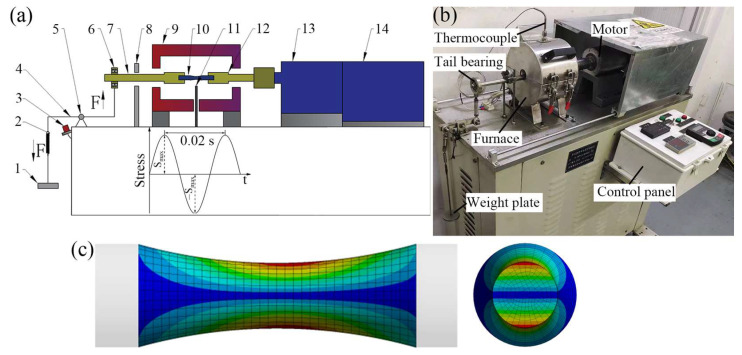
High-temperature RBF testing machine: (**a**) schematic diagram and structural compositions, (**b**) a picture of real product, (**c**) specimen stress nephogram. 1. Weights, 2. Spring, 3. Approach switch, 4. Loading lever, 5. Hinged support, 6. Tail bearing, 7. Left clamp, 8. Protective frame, 9. Heating furnace, 10. Specimen, 11. Thermocouple, 12. Right clamp, 13. Power shaft, 14. High-speed motor.

The high-temperature RBF testing machine is illustrated in Figure 5. The specimen underwent cyclic stress variations driven by a bending moment applied through weights. During the rotation of the RBF specimen, stress alternated between compressive and tensile at the same cross-sectional location, with a stress ratio (R) of −1 and a frequency of 50 Hz. The RBF test was terminated if the sample fractured or survived by 1 × 10^7^ cycles. RBF tests were conducted at temperatures of room temperature (RT) about 25 °C, 400 °C, and 650 °C (typical operational temperatures for engine exhaust valves). For high-temperature test, specimens were subjected to low-speed rotation and gradual heating within the furnace until temperature stabilization before testing.

### 2.3. Material Characterization

The microstructure of untreated and SMRT specimens was analyzed by using Optical microscope (OM, LeicaDMI8, Leica Camera AG, Wetzlar, Germany) and electron backscattered diffraction (EBSD, FEI Nova NanoSEM 230, FEI Company, Hillsboro, OR, USA). The variation of hardness along depth was measured using a Vickers microhardness tester (Veiyee HV-1000A, Shenzhen Miaozhun Instrument Equipment Co., Ltd., Shenzhen, China), with a load of 200 g and a dwell time of 10 s. Average hardness values and standard deviations were calculated based on three hardness measurements for each data point. Surface roughness Ra was measured using a roughness meter (Hommel W55, Hommel-Etamic GmbH, Jena, Germany). RS of the specimen was measured using an X-ray diffractometer (XRD, Smart Lab, Rigaku Corporation, Tokyo, Japan) by sin^2^*ψ* method, and the scanning parameters were set as: 48–53 degrees, speed 6° per minute, side-inclination method, parallel optical path. Then, the variation of RS along depth was measured by electrolytical removal of a thin surface layer and subsequent XRD measurement. Fractured specimens were cleaned in anhydrous ethanol using an ultrasonic cleaner, followed by observing the fracture surface of specimens using scanning electron microscope (SEM, FEI Quanta 250, FEI Company, Hillsboro, OR, USA).

## 3. Results and Discussion

### 3.1. Microstructure and Surface Roughness Ra

**Figure 6:**
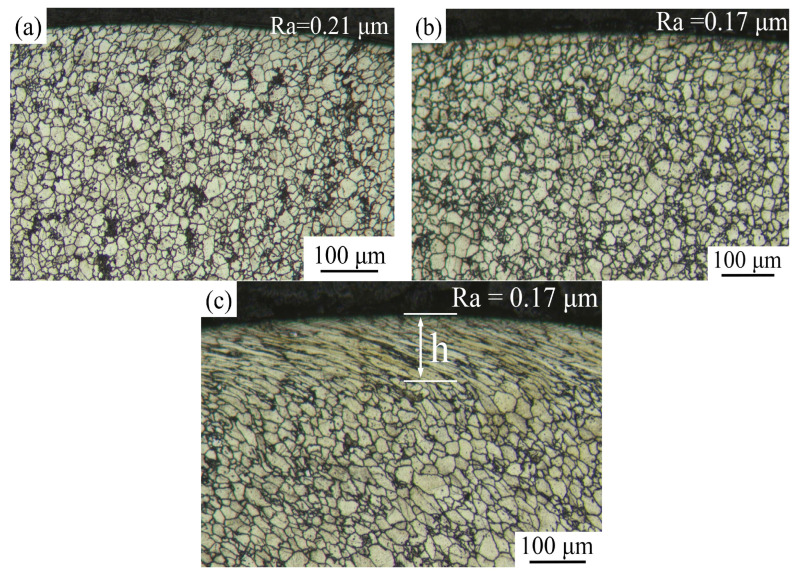
Cross-sectional microstructures of different specimens: (**a**) untreated, (**b**) 800-1 specimen, (**c**) 800-10 specimen.

SMRT specimens were labeled as 800-1 specimen (800 N with rolling 1 pass) and 800-10 specimen (800 N with rolling 10 passes), among others. This study focuses on investigating untreated, 800-1 and 800-10 specimen. The cross-sectional microstructure of the different status specimen, as shown in Figure 6, presents an austenitic structure and dispersed carbide. Severe plastic deformation has altered the surface layer of the specimens. 800-1 specimen exhibits less grain refinement, whereas 800-10 specimen exhibits elongated grains oriented along the rolling direction due to severe plastic deformation. With the assistance of the optical microscope, the deformation depth of 800-10 specimen is approximately 113 μm. This observation aligns with the findings reported by Wang et al. [8] and Liu et al. [21], which highlighted significant plastic deformation in the surface layer of SMRT specimens, while deformation depth correlated to grain refinement levels to some extent. Additionally, Ting et al. [22] observed that increasing the number of SMRT rolling passes can enhance the grain refinement effect. The initial surface roughness Ra of the turned specimens before SMRT was 0.21 μm. After SMRT, the surface roughness Ra was decreased to 0.17 μm.

**Figure 7:**
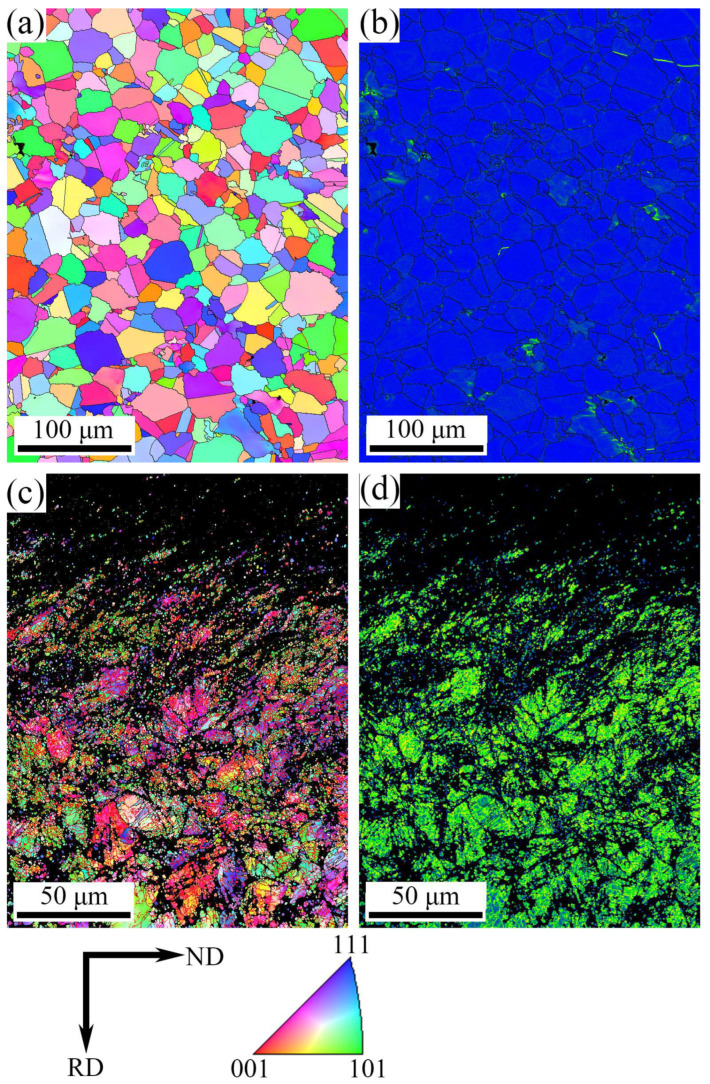
EBSD images: (**a**) IPF maps of untreated specimen, (**b**) KAM maps of untreated specimen, (**c**) IPF maps of 800-10 specimen, (**d**) KAM maps of 800-10 specimen.

Figure 7 shows EBSD images of the untreated and 800-10 specimen specimens, including IPF maps (Figure 7a,c) and Kernel average misorientation (KAM) maps (Figure 7b,d). The surface layer of the 800-10 specimen observed in Figure 7c,d exhibit dark areas in the EBSD images, similar to the findings reported by Carneiro et al. [11], which correspond to areas that cannot be resolved due to severe plastic deformation and the limitations of EBSD resolution. These unresolved zones were seen for other high-deformation manufacturing processes, such as cold spraying [23] or extrusion [24]. For example, Vaz et al. [23] also used EBSD technique to characterize the grain orientation and deformation behavior of cold spray additive manufacturing 316L material. The microstructures are EBSD maps based on band contrast information. They found that both cold spray additive manufacturing strategies in as-sprayed conditions present microstructures with a high deformation degree, leading to regions of low band contrast. In addition, Companhoni et al. [24] reported that metallic materials produced through equal channel angular extrusion may offer enhanced mechanical properties over classic thermomechanical processing like extrusion or rolling. During the microstructure characterization work, the EBSD could reveal that the microstructure was heterogeneous with a few large grains surrounded by a fine submicrometric structure, however, it was only partially resolved through this technique. Such fine structure could be described through automated crystal orientation mapping in TEM.

Compared to the untreated specimen, the IPF map shows a significant presence of sub-grain boundaries in the 800-10 specimen, with refined grains in the near-surface region and gradually transitioning into coarser grains towards the interior material (Figure 7c). As shown in KAM map of Figure 7b, the blue regions indicate lower local grain misorientations between the grains. In Figure 7d, yellow-green regions indicate the larger grain misorientations. Therefore, the 800-10 specimen exhibits larger local grain misorientations compared to the untreated specimen, indicating the presence of dense dislocations and evident dislocation pile-ups within the surface layer of 800-10 specimen. These results demonstrate that multiple passes of SMRT introduce cumulative plastic deformation, resulting in the formation of a GFS layer composed of refined grains and dislocations.

### 3.2. Residual Stress and Hardness Distribution

**Figure 8:**
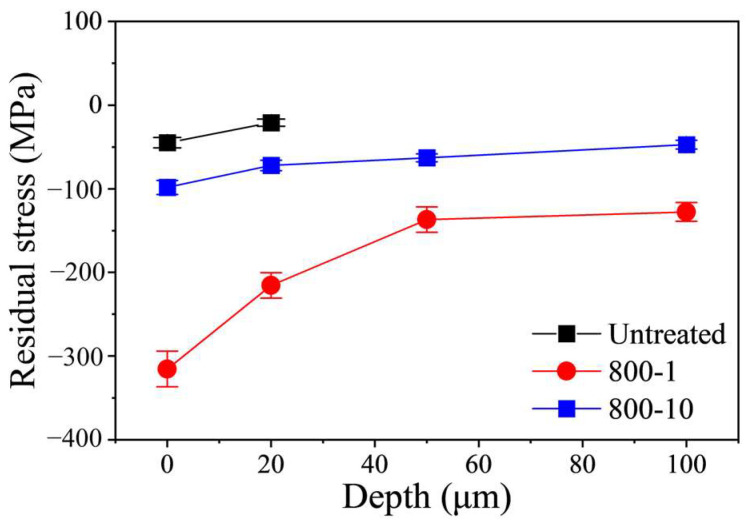
Variation of RS along depth.

Figure 8 illustrates the variation of RS measurement result along depth. The RS of each specimen reaches a maximum at the top surface and decreases as depth increases. For the 800-1 specimen, the RS value of top surface approximately was −315.4 MPa, while the RS value was only −98.4 MPa of 800-10 specimen. The RS of 800-10 specimen was significantly reduced, because the micro-defects caused by over-treatment relaxed the RS. Similar findings have been also found by Liu et al. [21]. They reported that the residual stress was markedly reduced with increased coverage rate or processing times of ultrasonic surface rolling treatment.

**Figure 9:**
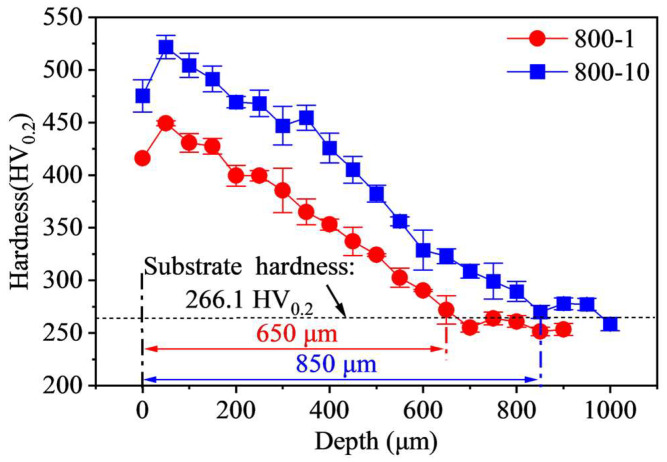
Variation of Vickers hardness along depth.

Figure 9 displays the variation of Vickers hardness along depth. It can be seen that untreated specimens exhibit an average hardness of 266.1 HV_0.2_, while SMRT specimens present a significant enhancement in hardness. The subsurface hardness of 800-1 specimen and 800-10 specimen are significantly higher, reaching 449.4 HV_0.2_ and 521.7 HV_0.2_ at a depth of 50 μm, representing an increase of 68.9% and 96.1%, respectively, compared to the untreated specimens. Furthermore, the hardened layer extends to depths of 650 μm for 800-1 specimen and 850 μm for 800-10 specimen. The 4Cr14Ni14W2Mo steel exhibits excellent plasticity. As the number of rolling passes increases, severe plastic deformation introduces compressive RS, dense dislocation, and increased grain density in the surface layer of the specimen. It is worth emphasizing that the depth of the hardened layer, as well as the extent of grain refinement, is not solely determined by the apparent plastic deformation depth of 113 μm.

### 3.3. Tensile Test Results

#### 3.3.1. Tensile Performance

**Figure 10:**
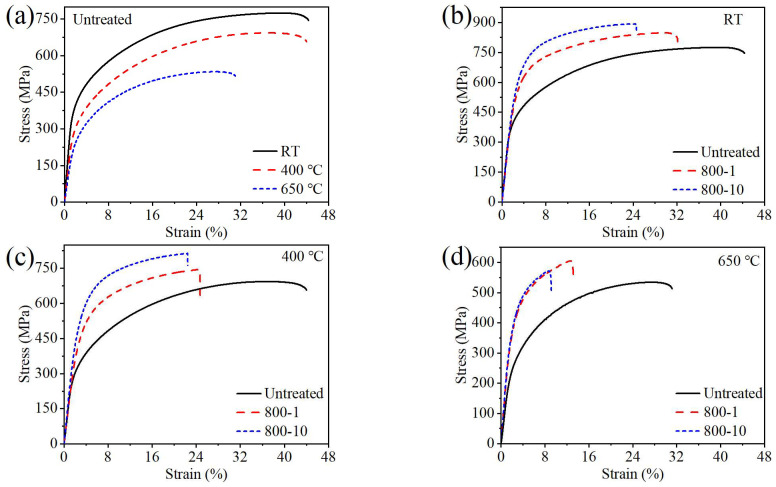
The comparison of tensile stress–strain curves at different temperatures: (**a**) untreated specimen, (**b**) RT, (**c**) 400 °C, (**d**) 650 °C.

The tensile stress-strain curves at different temperatures are shown in Figure 10, and the tensile performance is presented in Table 3. Compared to the results at RT, the tensile strength decreases from 774 MPa to 692 MPa (10.6%) at 400 °C and 533 MPa (31.1%) at 650 °C. Additionally, the plasticity remains a high level at 400 °C but decreases significantly at 650 °C.

The grain size of material significantly influences its yield strength, following the Hall–Petch relationship [25]. Compared to the untreated specimen, the strength of the 800-1 specimen and the 800-10 specimen increased while plasticity decreased at different testing temperatures. Specifically, the tensile strength of 800-1 specimen increased by 9.4% at RT, 7.5% at 400 °C, and 13.5% at 650 °C, with corresponding yield strength increases of 23.1%, 62.7%, and 51.9%. Similarly, the tensile strength of the 800-10 specimen increased by 15.2% at RT, 17.3% at 400 °C, and 7.1% at 650 °C, with respective yield strength increases of 33.4%, 50.6%, and 47.1%. SMRT enhanced the mechanical performance of the surface layer of material, therefore boosting its tensile strength [26]. At 650 °C, both specimens show a significant reduction in plasticity. Additionally, the strength of the 800-10 specimen was lower compared to the 800-1 specimen, which may be attributed to the influence of SMRT and high temperature, resulting in significantly lower plasticity and immediate fracture without deformation.

#### 3.3.2. Tensile Fracture Surfaces Analysis

**Figure 11:**
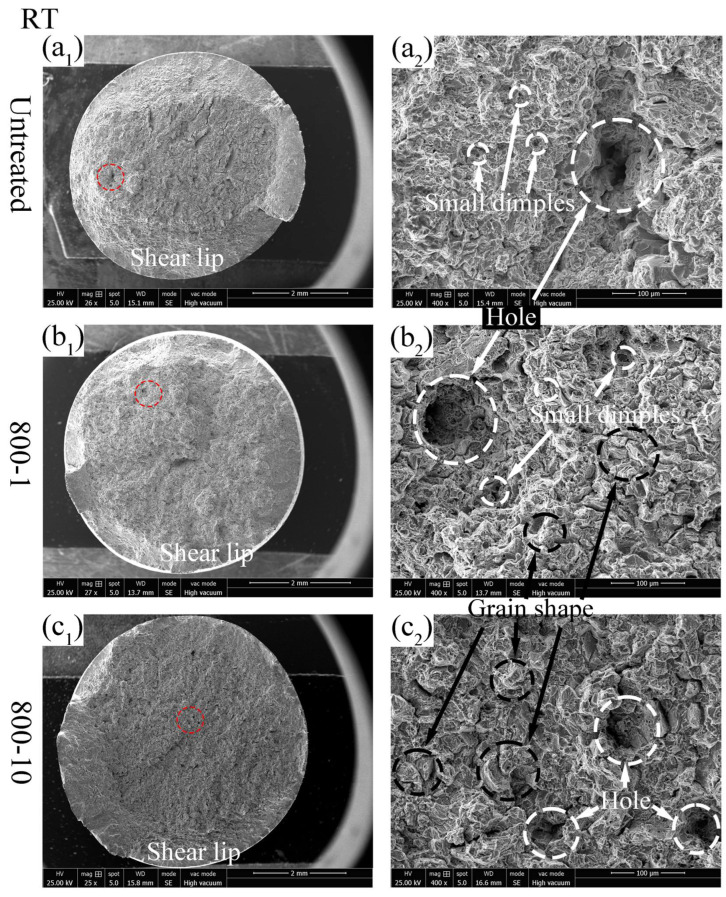
Tensile fracture surface of different specimens at RT: (**a_1_**,**a_2_**) untreated; (**b_1_**,**b_2_**) 800-1 specimen; (**c_1_**,**c_2_**) 800-10 specimen.

The tensile fracture surface depicted in Figure 11(a_1_–c_1_) shows the overall view of the fracture surface at RT. Figure 11(a_2_–c_2_) provides a detailed view of the fracture surface at RT. The fracture surface of the untreated specimen exhibits numerous small dimples and a larger hole (Figure 11(a_2_)), indicating ductile fracture behavior. The fracture surface of the 800-1 specimen displays a distinct grain-like appearance with some small dimples and a larger hole (Figure 11(b_2_)). Similarly, the fracture surface of the 800-10 specimen shows grain-like intergranular fracture with some small dimples and a noticeable reduction in hole depth (Figure 11(c_2_)). It could be summarized that the specimen exhibits more brittle fracture behavior as the number of rolling passes increases, due to the gradient fine structure introduced to the specimen surface, leading to the enhancement of tensile strength.

**Figure 12:**
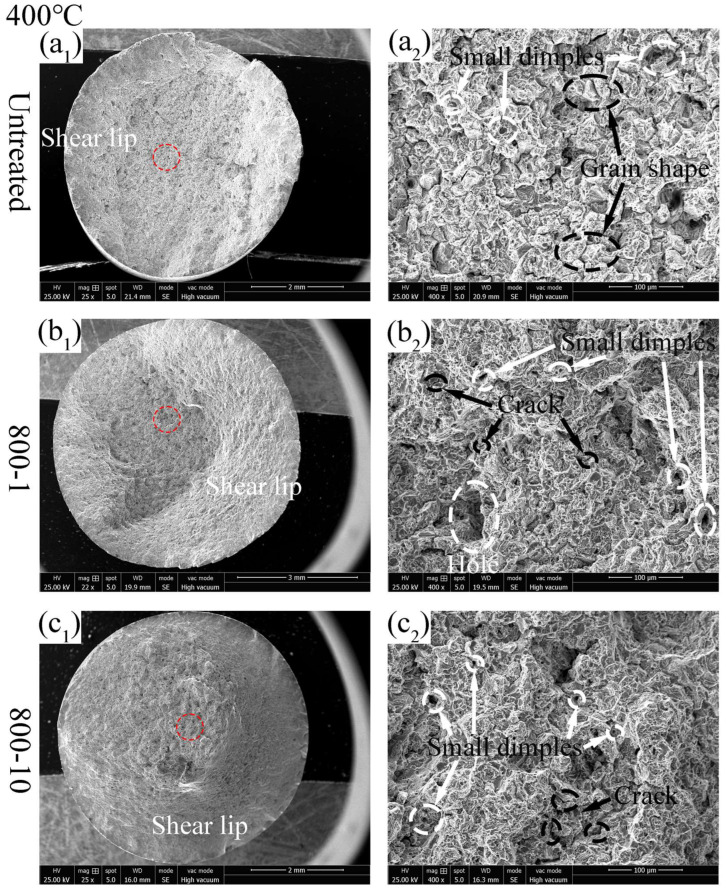
Tensile fracture surface of different specimens at 400 °C: (**a_1_**,**a_2_**) untreated; (**b_1_**,**b_2_**) 800-1 specimen; (**c_1_**,**c_2_**) 800-10 specimen.

At the tensile test temperature of 400 °C, as shown in Figure 12(a_1_–c_1_), it can be seen that the shear lip region of 800-1 specimen and 800-10 specimen has expanded compared to the untreated specimen, indicating increased material brittleness. This phenomenon correlates with the decreased section shrinkage rate of the SMRT specimen tested at 400 °C. As shown in Figure 12(a_2_–c_2_), all specimens exhibited a predominant brittle fracture behavior, with ductile fracture characteristics also present in varying degrees.

**Figure 13:**
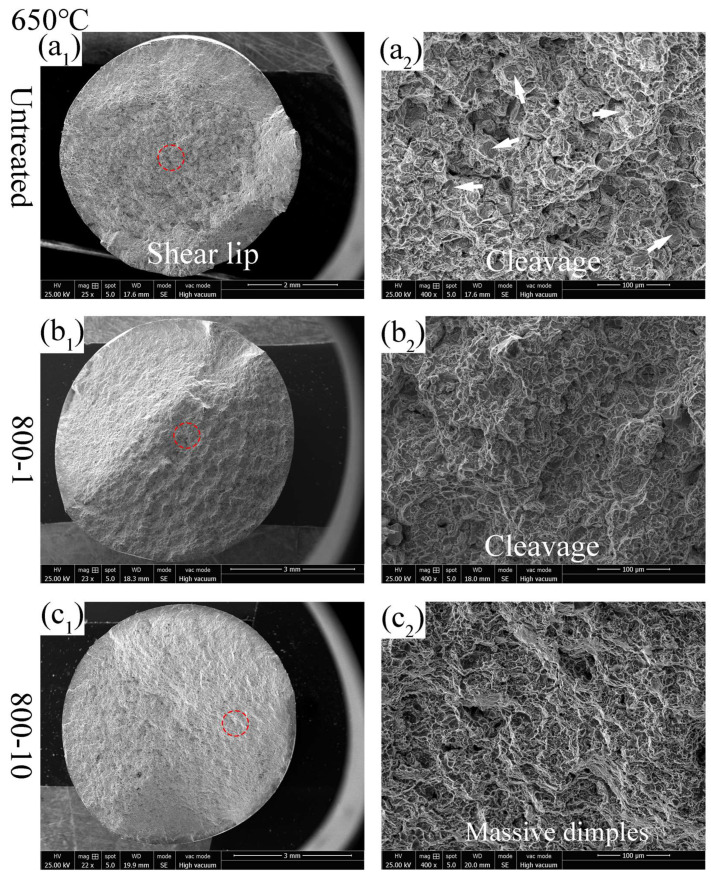
Tensile fracture surface of different specimens at 650 °C: (**a_1_**,**a_2_**) untreated; (**b_1_**,**b_2_**) 800-1 specimen; (**c_1_**,**c_2_**) 800-10 specimen.

At the tensile test temperature of 650 °C, the untreated specimen and the 800-1 specimen exhibit more cleavage fracture features (Figure 13(a_2_,b_2_)), indicating brittle fracture behavior. As shown in Figure 13(c_1_), it can be seen that the 800-10 specimen exhibits a smooth surface with an almost non-existent shear lip region, indicating high brittleness. Additionally, numerous small dimples observed in the 800-10 specimen (Figure 13(c_2_)) are conducive to crack propagation and rapid expansion. The high brittleness and numerous dimples caused the 800-10 specimen to fracture abruptly upon reaching local stress limits, bypassing uniform deformation and leading to a significant decrease in tensile strength at 650 °C (Figure 10d). At an elevated test temperature of 650 °C, residual stress relaxation occurred. For instance, Lai et al. [6] reported the residual stress relaxation results; they found that after tempering at 650 °C for 4 h, the compressive residual stress of an ultrasonic surface rolling treated specimen surface was relaxed from −803 MPa to −329 MPa. Furthermore, the grain size of the gradient fine structure was increased due to the elevated test temperature. Hence, the 800-1 and 800-10 specimens presented similar tensile strength and fracture surface characters.

### 3.4. Rotary Bending Fatigue Test Results

Fatigue is the process of localized damage in a component caused by cyclic loading or stress [27]. It is a cumulative effect resulting from the initiation, propagation, and eventual fracture of cracks within the component. The concept of fatigue strength can be explained from a physical perspective under constant amplitude loading conditions. Under alternating stresses, microcracks initiate within the material grains and propagate until halted by grain boundaries. If these grain boundaries are insufficiently strong, the microcracks can progress into macroscopic cracks, resulting in potential failure. Conversely, strong grain boundaries will suppress microcrack growth, known as non-propagating cracks.

#### 3.4.1. Rotary Bending Fatigue Performance

Based on the national standard, the fitting of the stress-life (S-N) curve uses a three-parameter power function fitting equation [28,29], as represented by Equation (1), where *S*_0_ is the fatigue limit parameter, β is the exponent of the S-N curve, and α is the fatigue strength coefficient.


(1) $$N{\left(S-S_{0}\right)}^{\;\beta }=\alpha$$

**Figure 14:**
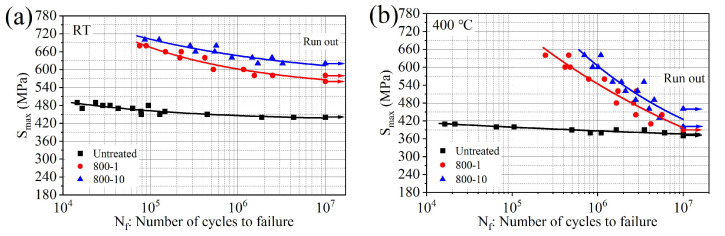

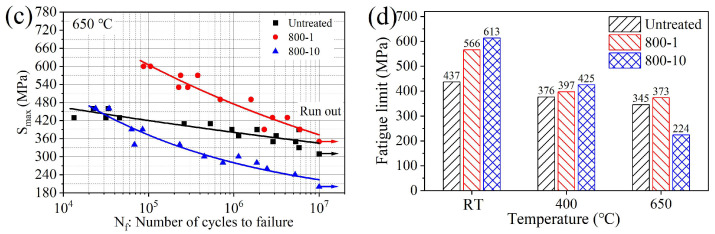
The comparison of RBF performance: (**a**) S-N curves at RT, (**b**) S-N curves at 400 °C, (**c**) S-N curves at 650 °C, (**d**) comparison of fatigue limit at 1 × 10^7^ cycles.

The RBF performance is shown in Figure 14 (where the stress corresponding to 1 × 10^7^ cycles is the fatigue limit). The fitted equations of S-N curves for different specimens at different temperatures are listed in Table 4. Meanwhile, the relevant R-squared parameters (coefficient of determination) are calculated and listed in the same table, respectively. It can be observed that the fatigue performance of untreated specimens decreases continuously with increasing temperature (Figure 14a–c). The fatigue limits are 376 MPa at 400 °C and 345 MPa at 650 °C, with a difference of 31 MPa, indicating a mere 8.2% decrease. This indicates that the material exhibits good fatigue performance at high temperatures.

At RT, as shown in Figure 14a,d, the fatigue limit of the 800-1 specimen increased from 437 MPa to 566 MPa (29.5%); for the 800-10 specimen, the fatigue limit increased to 613 MPa (40.3%). Compared to the untreated specimens, the improvement in fatigue limit is pronounced.

At 400 °C, compared to the 800-1 specimen, the fatigue performance of the 800-10 specimen shows further improvement (Figure 14b), influenced by GFS layer. The fatigue limit at 1 × 10^7^ cycles of 800-1 specimen increased from 376 MPa (untreated specimen) to 397 MPa (5.6%). For the 800-10 specimen, it further increased to 425 MPa (13.0%). It is indicated that the GFS layer could maintain reasonable performance at 400 °C.

At 650 °C, as shown in Figure 14c,d, compared to the untreated specimen, the fatigue limit at 1 × 10^7^ cycles of the 800-10 specimen exhibits a significant decrease; it decreased from 345 MPa to 224 MPa (−35.1%) (Figure 14d), and this fatigue performance degradation could be attributed the low elongation of the 800-10 specimen at 650 °C. It is associated with the tensile test results; it is found that the 800-10 specimen revealed a reduced elongation, while the fracture surface, characterized by numerous interconnected dimples, suggests a mechanism that may be conducive to rapid crack propagation (Table 3 and Figure 13(c_2_)). In general, the metallic materials with a high elongation can disperse local stress concentration through plastic deformation, thereby delaying the initiation of fatigue cracks. Similar to the findings of Wang et al., due to prolonged exposure of the specimen to high temperatures, the RS gradually diminishes, thereby affecting the resistance of material to fatigue stresses [30]. It could be inferred that the RS plays a significant role in fatigue strength for SMRT treated specimen, which also explains the increase in fatigue limit at 25 °C, while the increase in fatigue limit at high temperatures is not significant. A detailed analysis of the fracture surface from the RBF test will be presented in the following section.

#### 3.4.2. Fatigue Fracture Surfaces

**Figure 15:**
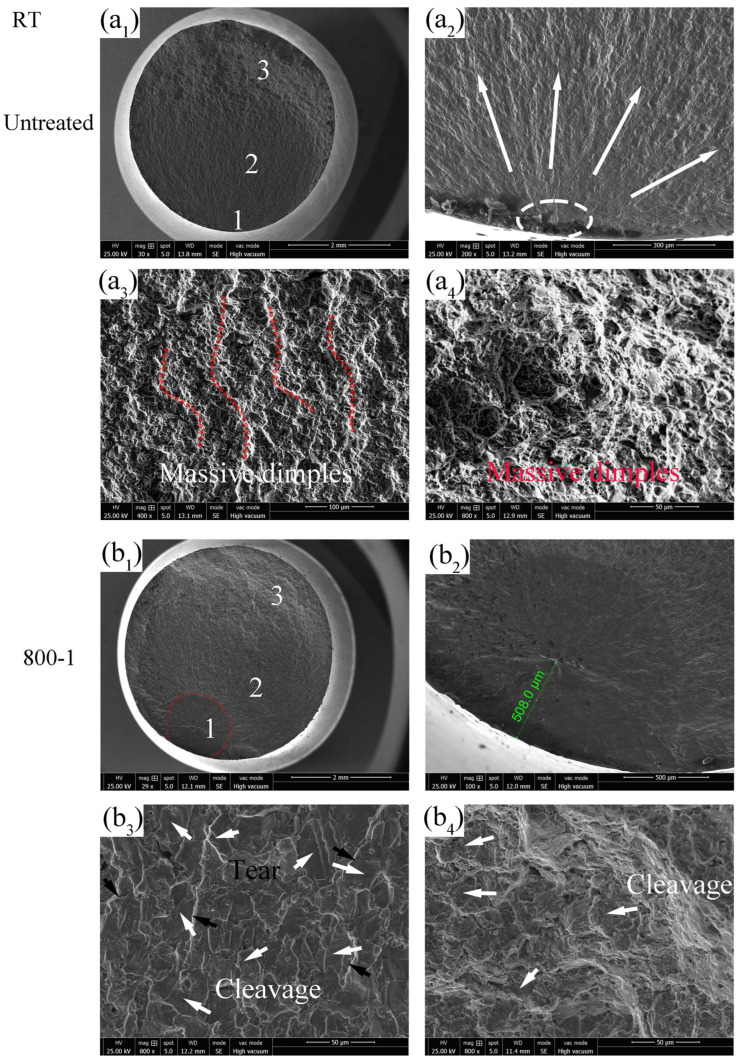
Fatigue fracture surface at RT: (**a_1_**–**a_4_**) σ = 470 MPa, N_f_ = 0.11 × 10^7^ cycles, (**b_1_**–**b_4_**) σ = 580 MPa, N_f_ = 0.24 × 10^7^ cycles.

The fatigue fracture surfaces at RT are shown in Figure 15, which can be divided into three typical regions: crack initiation zone (1), crack propagation zone (2), and instantaneous fracture zone (3), each specific zone is labeled as “1”, “2” and “3” in Figure 15(a_1_,b_1_), respectively. Fatigue cracks propagate sequentially along these three regions until the specimen fractures. The crack initiation zone is usually small, typically comprising only 2–5 grains, but it can account for up to 90% of the total fatigue life [31]. The crack in the untreated specimen initiates on the top surface and is characterized by a single-source initiation mechanism (Figure 15(a_2_)). In zone 1, distinct fan-shaped crack extension patterns are visible (propagating along the direction of the arrows), and the surface appears relatively smooth. Zone 2 in the untreated specimen exhibits numerous dimples and “ridges” consistent with the crack extension direction (Figure 15(a_3_)). These dimples are formed as voids created by internal plastic separation gradually enlarge under slip action, promoting crack propagation. As depicted in Figure 15(b_2_), it can be seen that the crack in the 800-1 specimen initiates from the subsurface, approximately 508 μm away from the top surface, which is indicative of a subsurface fracture mechanism. The transition of crack initiation mode was also reported by Lai et al. [6] and Sun et al. [32].

In general, fatigue fractures of components initiate from surface crack. However, SMRT introduces a surface modified GFS layer consisting of refined grains, a hardened layer, and RS, which could significantly increase the surface layer performance of material, reducing the likelihood of crack initiation from the top surface, thereby improving the fatigue performance [33,34]. Zone 2 in the 800-1 specimen shows more cleavage planes and tear features (Figure 15(b_3_)), while zone 3 in the 800-1 specimen also displays more cleavage planes (Figure 15(b_4_)). The increase in cleavage fracture features indicates a tendency towards brittle fracture failure mechanism.

**Figure 16:**
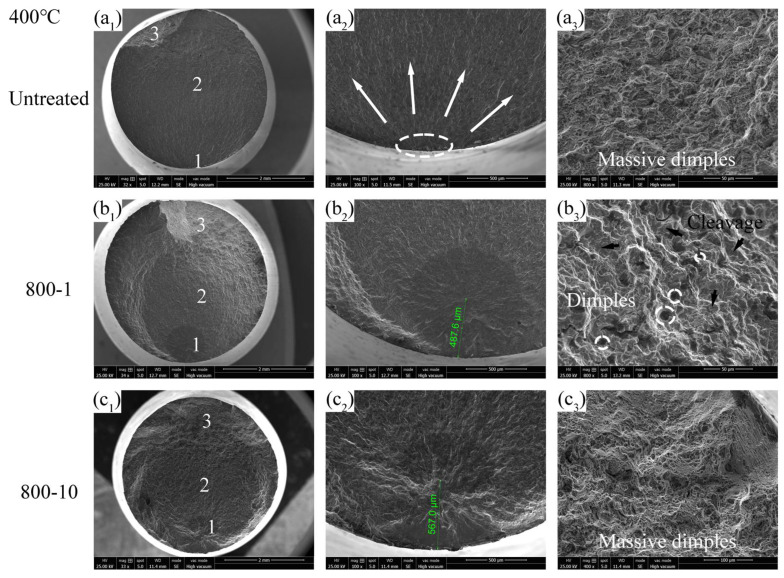
Fatigue fracture surface at 400 °C: (**a_1_**–**a_3_**) σ = 380 MPa, N_f_ = 0.08 × 10^7^ cycles, (**b_1_**–**b_3_**) σ = 520 MPa, N_f_ = 0.17 × 10^7^ cycles, (**c_1_**–**c_3_**) σ = 520 MPa, N_f_ = 0.29 × 10^7^ cycles.

The fatigue fracture surfaces at 400 °C are shown in Figure 16 and also divided into three regions, each specific zone is labeled as “1”, “2” and “3” in Figure 16(a_1_,b_1_,c_1_), respectively. As at 400 °C, the crack of the untreated specimen initiates on the top surface and propagates in a fan-shaped pattern, indicating surface fracture (Figure 16(a_2_)). Zone 3 in the untreated specimen exhibits numerous ductile dimples (Figure 16(a_3_)), representing ductile fracture characteristics. As shown in Figure 16(b_1_), zone 3 in the 800-1 specimen appears larger compared to the untreated specimen, suggesting higher brittleness. The crack initiation position of the 800-1 specimen is approximately 487.6 μm from the top surface (Figure 16(b_2_)), which is less than the crack initiation distance observed at RT (Figure 15(b_2_)). Additionally, zone 3 in the 800-1 specimen displays cleavage planes and some dimples (Figure 16(b_3_)). It could be inferred that prolonged exposure to high temperatures gradually causes the RS to relax, resulting in a decrease in the performance of the surface layer and a weakening of the microscopic barriers that resist crack initiation and propagation. Concurrently, the high temperature also slightly restores the plasticity of the specimen, resulting in more ductile fracture features compared to RT. The crack initiation position of 800-10 specimen is approximately 567 μm from the top surface (Figure 16(c_2_)), which exhibits an increased transfer distance compared to the 800-1 specimen, attributed to a significantly thicker hardened layer and GFS layer (Figure 6), effectively enhancing fatigue performance (Figure 14c). Zone 3 in the 800-10 specimen exhibits numerous dimples (Figure 16(c_3_)), demonstrating ductile fracture behavior.

**Figure 17:**
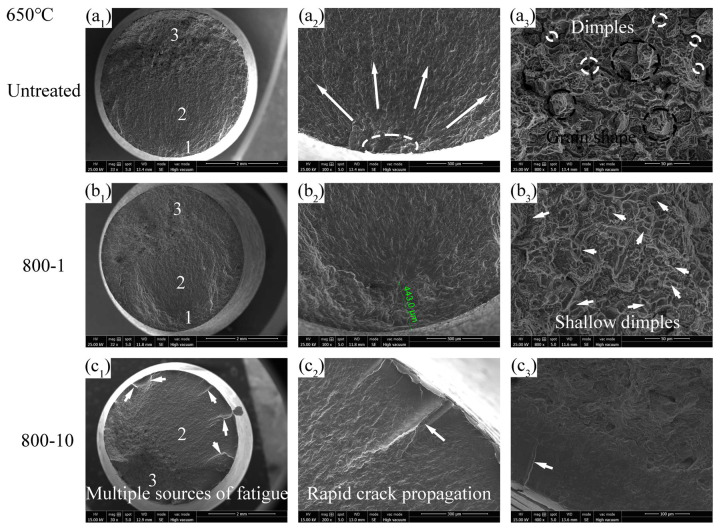
Fatigue fracture surface at 650 °C: (**a_1_**–**a_3_**) σ = 390 MPa, N_f_ = 0.58 × 10^7^ cycles, (**b_1_**–**b_3_**) σ = 430 MPa, N_f_ = 0.28 × 10^7^ cycles, (**c_1_**–**c_3_**) σ = 480 MPa, N_f_ = 0.005 × 10^7^ cycles.

The fatigue fracture surfaces at 650 °C are shown in Figure 17. Zone 1 in the untreated specimen indicates a surface fracture mechanism (Figure 17(a_2_)). In zone 3 in the untreated specimen (Figure 17(a_3_)), dimples and intergranular fracture features are observed, suggesting an increased likelihood of brittle fracture of the material at 650 °C, which is consistent with the tensile fracture behavior. The 800-1 specimen shows subsurface fracture mechanism, with the disappearance of “fish eyes” (Figure 17(b_1_)). Additionally, the crack initiation position of the 800-1 specimen is further reduced to approximately 443 μm from the specimen surface (Figure 17(b_2_)). Finally, zone 3 in the 800-1 specimen exhibits ductile fracture behavior (Figure 17(b_3_)). These phenomena could be due to the fact that, similar to 400 °C, the high temperature weakens the microscopic barriers that resist crack initiation and propagation and slightly restores the plasticity of the specimen. Additionally, the dissipation of the RS increased the impact of fatigue tensile stress, resulting in dimples being more prone to formation and interconnection under tensile stress conditions. This could explain why, because the original RS of the 800-10 specimen is less than that of the 800-1 specimen, the fracture surface of the 800-10 specimen exhibits more dimples compared to the 800-1 specimen at 400 °C.

The 800-10 specimen exhibits a distinctive fracture surface at 650 °C, characterized by multi-source fractures and stripes resulting from the rapid propagation of cracks (Figure 17(c_2_)). The smooth edge of the fracture surface of the 800-10 specimen can be observed, indicating the presence of a modified surface layer (Figure 17(c_3_)). However, the high brittleness of the 800-10 specimen at 650 °C results in the decrease in its fatigue performance.

## 4. Conclusions

In this study, surface mechanical rolling treatment (SMRT) with multiple passes was utilized on 4Cr14Ni14W2Mo steel to improve its high-temperature rotary bending fatigue (RBF) resistance. The fatigue and fracture mechanisms were analyzed. The main conclusions of the study are as follows:

(1) Multiple rolling passes significantly refined the surface layer of 4Cr14Ni14W2Mo steel, resulting in the formation of a gradient fine structure layer composed of fine grains and dislocations. Additionally, the specimen after SMRT showed lower surface roughness Ra and higher compressive RS and hardness of the material surface layer.
(2) The tensile strength of SMRT specimens increased while their plasticity decreased. It is noteworthy that at 650 °C, specimens with 10 rolling passes exhibited extremely high brittleness, characterized by flat fracture surfaces, resulting in a reduction in tensile strength compared to specimens with one rolling pass.
(3) The RBF fatigue limits were 437 MPa at RT, 376 MPa at 400 °C, and 345 MPa at 650 °C. Employing SMRT with one pass, the fatigue limit increased to 566 MPa (29.5%) at RT, but limited improvement was observed at high temperatures of 400 °C and 650 °C.
(4) Employing SMRT with 10 passes, the fatigue limit increased to 613 MPa (40.3%) at RT. The fatigue limit increased to 425 MPa (13.0%) at 400 °C; however, it decreased to 224 MPa (−35.1%) at 650 °C. This decrease is due to the high brittleness of the specimen, which resulted in the rapid initiation and propagation of cracks.
(5) The improvement of RBF fatigue strength of SMRT specimens could be attributed to a modified surface layer that consists of beneficial compressive residual stress, higher hardness, and gradient fine structure layer in the severe plastic deformation layer. Consequently, crack initiation and extension are hindered, resulting in the transfer of crack initiation from the top surface to the subsurface.

## Figures and Tables

**Table 1:** Chemical compositions of the 4Cr14Ni14W2Mo steel (in wt.%).

C	Si	Mn	P	S	Ni	Cr	Mo	W	Fe
0.43	0.396	0.55	0.036	0.002	13.35	13.57	0.321	2.09	Bal.

**Table 2:** SMRT processing parameters.

Load (N)	Vs (r/min)	Vr (mm/r)	The Number of Rolling Passes
800	160	0.08	0, 1, 10

**Table 3:** Tensile performance of different specimens.

Specimen	Temperature (°C)	Tensile Strength (MPa)	Yield Strength (MPa)	Section Shrinkage (%)
Untreated	RT	774	350	39.3
	400	692	255	36.8
	650	533	210	29.3
800-1	RT	847	431	36.8
	400	744	415	17.7
	650	605	319	10.2
800-10	RT	892	467	23.3
	400	812	384	15.5
	650	571	309	6.7

**Table 4:** The fitted equations of different specimens at different temperatures.

Temperature	Specimen	Fitted Equation	R-Squared (Coefficient of Determination)
RT	Untreated	${N(S-428.8326)}^{3.4014}=1.4795\times 10^{10}$	0.8212
RT	800-1	${N(S-529.8837)}^{3.2960}=1.3210\times 10^{12}$	0.9329
RT	800-10	${N(S-562.4463)}^{4.5641}=6.1242\times 10^{14}$	0.8688
400	Untreated	${N(S-336.5098)}^{10.2145}=2.0631\times 10^{23}$	0.8634
400	800-1	$N{\left(S-0.00004587\right)}^{7.2674}=7.5834\times 10^{25}$	0.9039
400	800-10	$N{\left(S-179.9923\right)}^{4.1876}=1.0108\times 10^{17}$	0.8620
650	Untreated	$N{\left(S-0.0001711\right)}^{23.3831}=2.2088\times 10^{66}$	0.8029
650	800-1	$N{\left(S-0.0001062\right)}^{9.5147}=2.9394\times 10^{31}$	0.9106
650	800-10	$N{\left(S-133.6100\right)}^{4.7237}=1.6978\times 10^{16}$	0.9318

## Data Availability

The original contributions presented in this study are included in the article. Further inquiries can be directed to the corresponding author.

## Abbreviations

SMRT	Surface mechanical rolling treatment
GFS	Gradient fine structure
SEM	Scanning electron microscope
XRD	X-ray diffractometer
RBF	Rotary bending fatigue
EBSD	Electron backscattered diffraction
KAM	Kernel average misorientation
RS	Residual stress
